# Huoxue-Jiangtang decoction ameliorates type 2 diabetes in high-fat diet and streptozotocin-induced rats

**DOI:** 10.3389/fphar.2025.1675295

**Published:** 2025-10-17

**Authors:** Maosheng Lee, Yanyan Tan, Huilin Li, Deliang Liu, Hengxia Zhao

**Affiliations:** ^1^ The Fourth Clinical Medical College, Guangzhou University of Chinese Medicine, Guangzhou, China; ^2^ Department of Endocrinology, Shenzhen Traditional Chinese Medicine Hospital, Shenzhen, China

**Keywords:** type 2 diabetes, insulin resistance, traditional Chinese medicine, Huoxue-Jiangtang decoction, AKT, GLUT4

## Abstract

**Introduction:**

A traditional Chinese herbal decoction, Huoxue-Jiangtang decoction (HXJT), has been clinically prescribed to patients with type 2 diabetes mellitus (T2DM) to improve hyperglycemia and hyperlipidemia for many years. However, the potential mechanisms underlying its anti-diabetic effects remain unclear.

**Methods:**

The aim of this study was to explore the anti-diabetic effects and underlying molecular mechanisms of HXJT in T2DM rats, which were fed with a high-fat diet (HFD) and subsequently induced with streptozotocin (STZ). HPLC-MS analysis was performed to characterize the chemical composition of HXJT and serve as a quality control measure. The T2DM rats were treated with metformin or HXJT for 8 weeks.

**Results and discussion:**

Treatment with HXJT significantly reduced hyperglycemia and improved insulin resistance in T2DM rats, as revealed by multiple assessments, including fasting blood glucose (FBG), glucose tolerance, fasting insulin levels, homeostasis model assessment of insulin resistance, insulin sensitivity index analysis, and histological examination of pancreas islets. HXJT treatment decreased blood lipid profile, including total cholesterol, low-density lipoprotein cholesterol, and triglycerides, although it did not change the rats’ body weight. The Western blot results indicated that HXJT reversed the downregulation of AKT and PI3K and markedly increased glucose transporter type 4 (GLUT4) in skeletal muscles. Moreover, the levels of glycogen synthetase (GS), hexokinase, superoxide dismutase (SOD), glycogen, and muscle glycogen in the HXJT group significantly increased relative to those in untreated T2DM group, while TNF-α levels decreased observably. In conclusion, HXJT improves insulin resistance, enhances insulin sensitivity, and helps preserve glucose homeostasis. The potential molecular mechanisms are related to the activation of PI3K/AKT and GLUT4 in skeletal muscles, either directly or indirectly.

## 1 Introduction

According to the eighth edition of the Global Diabetes Survey released by the International Diabetes Federation (Cho et al., 2018), the number of diabetes mellitus (DM) patients worldwide has reached 450 million; in China, the number is 114 million, with type 2 diabetes mellitus (T2DM) accounting for more than 90% of diabetes patients. The all-cause mortality rate of patients with diabetes is approximately twice that of the general population (Gnatiuc et al., 2018). All situations indicate a large increase in absolute costs from $1.32 trillion in 2015 to $2.25 trillion in 2030 (2.18–2.34) under the baseline scenario (Bommer et al., 2018). DM is a non-communicable chronic disease that still has a long way to go before a cure is found; what we are doing is slowing down the progression and reducing the complications of symptoms. Its possible pathogenesis is complex; DM is a metabolic disease characterized by *β*-cell dysfunction and insulin resistance (Kahn et al., 2014) and is associated with inflammation resulting from overnutrition or other environmental factors (Donath and Shoelson, 2011; Shoelson and Goldfine, 2009).

Metformin is an oral anti-hyperglycemic drug and is most widely used in clinical practice; its anti-diabetic effect is mainly attributed to its action on the liver via AMPK activation (Rena et al., 2017). However, metformin treatment is commonly associated with gastrointestinal adverse effects, and long-term metformin use is associated with clear evidence of low vitamin B12 levels (Niafar et al., 2015), which may cause diabetic neuropathy or carry an increased risk of complications, including hyperhomocysteinemia, peripheral neuropathy, and megaloblastic anemia (Singh et al., 2013). When these side effects occur, most patients in China turn to traditional Chinese medicine (TCM) for supplementary treatment. TCM has been developed for the treatment of numerous diseases for many years. Huoxue-Jiangtang decoction (HXJT) is an herbal formula composed of eight Chinese herbs and was specifically developed for the prevention and treatment of T2DM. *Astragali Radix* (Kai et al., 2015), *Ophiopogonis Radix* (Chen et al., 2016), and *Carthamus tinctorius* (Asgary et al., 2012), which are the main components of HXJT, have significant effects on ameliorating blood glucose and lipid levels, and HXJT treatment has achieved a good curative effect in the treatment of patients with T2DM in China. However, its mechanisms of anti-diabetic effects remain unclear. Moreover, *Rehmanniae Radix*, the main component of HXJT, contains catalpol as its major chemical constituent, which may improve high-fat diet (HFD)-induced insulin resistance in mice by reducing tissue inflammation and inhibiting the NF-κB and JNK pathways (Zhou et al., 2015). Astragaloside IV has antioxidant (Huang et al., 2016) and hypoglycemic effects, which may be attributed to inhibiting hepatic glycogen phosphorylase and glucose-6-phosphatase activities (Lv et al., 2009). Therefore, we hypothesized that the anti-diabetic effects of HXJT are mainly attributable to the combined actions on multiple targets, including anti-inflammatory effects, mitigation of oxidative stress, and regulation of glycolipid metabolism in the liver and skeletal muscles.

Animal models that replicate the pathology of human T2DM are essential for evaluating the efficacy of preclinical drugs for T2DM. Feeding with HFD can develop insulin resistance, but not distinctly hyperglycemia or diabetes (Tanaka et al., 2007; Flanagan et al., 2008). Injection with a low dose of streptozotocin (STZ) induces a mild impairment of insulin secretion, which is similar to the feature of the later stage of T2DM (Szkudelski, 2012). Thus, the T2DM rat model, which is fed an HFD for 4–8 weeks followed by intraperitoneal injection of a low dose of STZ (30–40 mg/kg), closely simulates the natural history of human T2DM (Srinivasan et al., 2005). Moreover, this T2DM rat model has been widely used to study the efficacy of antidiabetic drugs (Sahin et al., 2013; Jiang et al., 2018).

Therefore, the objective of this study was to explore the efficacy and potential molecular mechanisms of HXJT in T2DM rats by feeding them with an HFD and treating them with STZ. While verifying the efficacy of HXJT and metformin, we explored how HXJT plays an anti-diabetic role in T2DM rats.

## 2 Materials and methods

### 2.1 HXJT preparation and quality control

**FIGURE 1:**
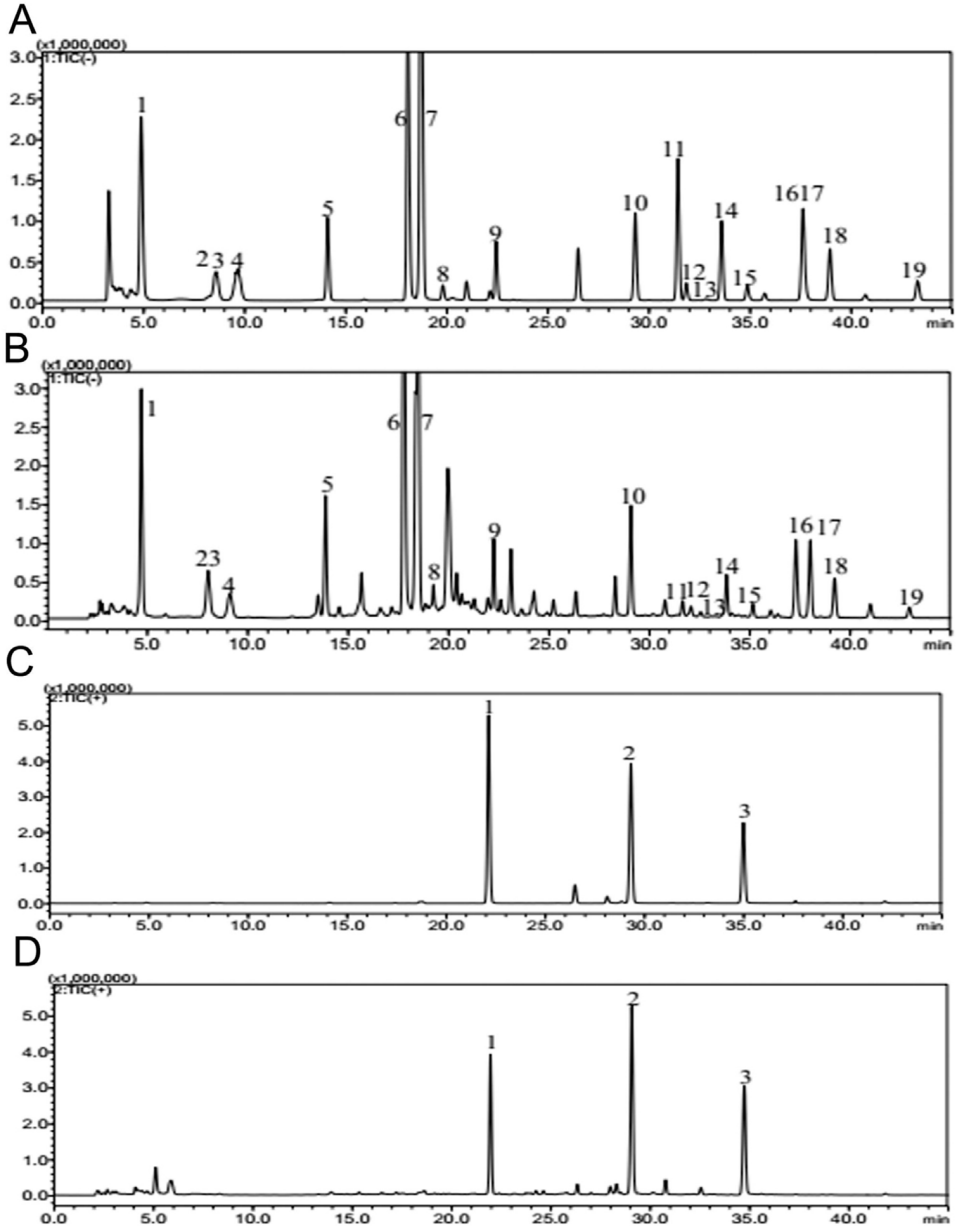
Quality control of HXJT through HPLC-MS. Total flowchart of reference negative ions **(A)**, total flowchart of HXJT-negative ions **(B)**, including 19 peaks: 1. catalpol; 2. aucubin; 3. gallic acid; 4. rehmannioside D; 5. ajugol; 6. hydroxysafflor yellow A; 7. amygdalin; 8. echinacoside; 9. verbascoside; 10. ononin; 11. astragaloside; 12. astragaloside III; 13. sprengerinin C; 14. astragaloside II; 15. isoastragaloside II; 16. rhein; 17. astragaloside I; 18. isoastragaloside I; and 19. emodin. Total flowchart of reference positive ions **(C)** and total flowchart of HXJT-positive ions **(D)**, including three peaks: 1. calycosin-7-O-B-D-glucoside; 2. calycosin; and 3. formononetin.

Huoxue-Jiangtang decoction contains eight herbs, and the proportion of each herb is demonstrated in Table 1. All botanical names can be verified and fully confirmed using the following portal: http://mpns.kew.org/mpns-portal/?_ga=1.111763972.1427522246.1459077346. The herbs used in our study were provided by Kangmei Pharmaceutical Co., Ltd, Shenzhen city, China (Lot no. XSKP2019022445), and the batch numbers of the respective herbs are presented in Table 1. The plant materials were identified by Prof. Shangbin Zhang based on their morphological features, and the voucher specimens were deposited in the herbarium of the Pharmaceutical Department, Shenzhen Traditional Chinese Medicine Hospital. The quality was controlled by the Shenzhen Key Laboratory of Hospital Chinese Medicine Preparation, Shenzhen Traditional Chinese Medicine Hospital. Quality control of all materials was authenticated based on the Chinese Pharmacopoeia (National Pharmacopoeia Commission, 2015). HXJT was prepared according to the manufacturing process of the native FDA in China. In brief, all herbs of HXJT were weighed and extracted in boiling water twice for 1 hour. Then, the combined filtrates were evaporated at room temperature to a final density of 2 g/mL and preserved at 4 °C until use. The HPLC-MS technique was performed to ensure the quality of the HXJT, as shown in Table 2 and Figure 1. Metformin was provided by Shiguibao (Shiguibao Co., Lt, China). HPLC-grade formic acid was purchased from TEDIA (Fairfield, OH, United States). Deionized water was obtained using a Milli-Q Ultrapure Water System (Millipore, Merck, Germany). HPLC analysis was conducted on a Shimadzu LC-20AT HPLC System equipped with an LC workstation CBM-10A VP Plus (Shimadzu, Japan).

**TABLE 1:** Composition and proportion of every herb in HXJT.

Plant family	Botanical name	Herbal name	Chinese name	Batch number	Specimen number	Dosage
Scrophulariaceae	*Rehmannia glutinosa* (Gaertn.) DC.	Rehmanniae Radix	Shengdi	181200019	181200019-02	30 g
Leguminosae	*Astragalus mongholicus* Bunge	Astragali Radix	Huangqi	181200171	181200171-01	30 g
Liliaceae	*Ophiopogon japonicus* (Thunb.) Ker Gawl	Ophiopogonis Radix	Maidong	181105081	181105081-03	15 g
Caryophyllaceae	*Pseudostellaria heterophylla* (Miq.) Pax	Pseudostellariae Radix	Taizishen	181000361	181000361-05	30 g
Asteraceae	*Carthamus tinctorius* L	Carthamus tinctorius	Honghua	181102711	181102711-04	12 g
Dioscoreaceae	*Dioscorea polystachya* Turcz	Dioscorea polystachya	Shanyao	1901101251	1901101251-06	12 g
Rosaceae	*Prunus persica* (L.) Batsch	Semen Persicae	Taoren	181001851	181001851-07	10 g
Polygonaceae	*Rheum officinale* Baill	Radix et Rhizoma Rhei	Dahuang	181000099	181000099-08	5 g

**TABLE 2:** Contents of the main identified compounds in the HXJT extract.

Herb name	Compound	Mass concentration (μg/g)
Astragali Radix	Astragaloside	4.35
	Calycosin-7-O-β-D-glucoside	328.27
	Calycosin	43.77
	Formononetin	18.8
	Astragaloside I	55
	Isoastragaloside I	50.16
	Isoastragaloside II	9.74
	Astragaloside II	6.45
	Astragaloside III	7.79
	Ononin	48.68
Rehmanniae Radix	Catalpol	**1,249.57**
	Verbascoside	96.87
	Echinacoside	5.13
	Aucubin	8.85
	Rehmannioside D	590.73
	Ajugol	267.21
Carthamus tinctorius	Hydroxysafflor yellow A	**1,672.07**
Radix et Rhizoma Rhei	Rhein	278.48
	Emodin	11.49
	Gallic acid	272.5
Semen Persicae	Amygdalin	**1,338.46**
Ophiopogonis Radix	Sprengerinin C	1.8

Hydroxysafflor yellow A, Amygdalin and Catalpol, represent the top three active constituents with the highest concentrations in HXJT.

### 2.2 Animal experiment

Six-week-old specific pathogen-free (SPF) male Wistar rats (weighted, 130 ± 20 g) were provided by the Guangdong Medical Laboratory Animal Center (Guangzhou City, China). All rats were kept in an SPF animal laboratory (12 h light/dark cycle, 20 °C, and 50%–70% humidity) with free access to water and food. T2DM rats were induced by feeding an HFD for 4 weeks, followed by treatment with a low dose of STZ (35 mg/kg) (Sigma Chemical Co. St.Louis, MO, United States) dissolved in 0.1 M citric acid–sodium citrate buffer (pH = 4.4) (Srinivasan et al., 2005). The formula of the HFD includes protein (26.2 gm%, 20 kcal%), carbohydrate (26.3 gm%, 20 kcal%), and fat (34.9 gm%, 60 kcal%); its detailed formula is provided in Supplementary Table S1. The HFD was a modified model based on the Van Heek L series 60% high-fat obesity model diet (Code D12492, Guangdong Medical Laboratory Animal Center Co., Ltd.) and was prepared according to the AIN-93 standard. Two weeks after treating with STZ, rats with fasting blood glucose (FBG) levels exceeding 11.1 mmol/L were validated as T2DM models (Lu et al., 2016). After modeling, T2DM rats were daily orally administered with HXJT (7.78 g/kg). The daily dose of HXJT for human use is 144 g of crude drugs. After lyophilization, the 144 g crude drug dose yields 49 g of HXJT-lyophilized powder. Based on the human-to-rat dose conversion factor (6.3), the corresponding dose for rats is 7.78 g/kg. Each 1 mL of the HXJT solution contains 1.47 g of the lyophilized powder. Metformin (90 mg/kg) or normal saline was administered for 8 weeks. The human dose of metformin was 1,000 mg (70 kg) per day (14.29 mg/kg), according to the human-to-rat dose conversion (6.3), and the corresponding dosage administered to rats was 90 mg/kg. Ten rats fed with a standard chow diet (5% fat) were also treated with normal saline as the normal control group. All rats were anesthetized using sodium pentobarbital, and blood was sampled from the aorta abdominalis at the end of the treatment. Pancreatic tissues were dissected and immersed in 10% neutral-buffered formalin for further studies.

All animal procedures were approved by the Institutional Animal Care and Use Committee of Tsinghua University and conducted in accordance with the National Institute of Health guidelines on animal ethics, and all efforts were made to minimize the animals’ suffering.

### 2.3 Biomedical analysis

The body weight, food consumption (kcal/g), and water consumption (mean) were monitored weekly. FBG was measured weekly using a glucometer (Roche Diagnostic Products Co. Ltd., Shanghai City, China), and blood samples were collected from the tail vein of the 12-h-fasted rats. An oral glucose tolerance test (OGTT) was conducted after 8 weeks of administration; the background level of FBG was defined as the FBG value at the eighth week. In short, OGTT was conducted in 12-h-fasted rats by measuring tail vein blood glucose levels after the administration of the glucose solution (2 g/kg, Sigma Aldrich, United States) by superalimentation at the time points of 0, 30, 60, and 120 min. The area under the curve (AUC) of OGTT was calculated.

Fasting blood insulin (FINS), total serum cholesterol (TC), triglycerides (TGs), high-density lipoprotein cholesterol (HDL-C), low-density lipoprotein cholesterol (LDL-C), leptin (LEP), adiponectin (APN), aspartate aminotransferase (AST), alanine aminotransferase (ALT), and free fatty acids (FFAs) were evaluated using a rat enzyme-linked immune sorbent assay (ELISA) kit, according to the manufacturer’s instructions (Beijing Solarbio Science and Technology Co., Ltd. Beijing, China; BioSino Bio-Technology and Science Inc. Beijing, China; Wuhan Mershack Biotechnology Co. Ltd., Wuhan, China). Glycogen synthetase (GS), hexokinase, superoxide dismutase (SOD), and NADP-malic dehydrogenase levels were determined using the WST-8 method. Hepatic glycogen levels in the liver and muscle glycogen levels in skeletal muscles were detected using the anthrone method, according to the manufacturer’s instructions (Solarbio Science and Technology Co., Ltd. Beijing, China). Insulin resistance, evaluated by the homeostatic model assessment of insulin resistance (HOMA-IR) (Hanley et al., 2002), was calculated using the formula: HOMA-IR = FINS (mU/L) × FBG (mmol/L)/22.5. The pancreatic islet function, assessed by the homeostasis model assessment of β-cell function (HOMA-β), was calculated using the formula: HOMA-*β*=(20 × FINS mU/mL)/(FBG mmol/L−3.5). The modified β-cell function index (MBCI) was calculated using the formula: MBCI = (FINS mU/mL × FBG mmol/L)/(BG2h + BG1h-2FBG), which was also selected to evaluate β-cell function. The formula to assess the insulin sensitivity index (ISI) is as follows: ISI = Ln [1/(FBG*FINS)] (Cederholm and Wibell, 1990).

### 2.4 Histological and immunohistochemical analyses

After 48 h of fixation, pancreatic tissues were prepared as paraffin sections of 3.5 µm, stained with hematoxylin–eosin (HE), and examined using a fluorescence microscope (Nikon Eclipse E100; Nikon, Tokyo, Japan).

The pancreatic tissues were fixed in 4% paraformaldehyde, deparaffinized in xylene, and rehydrated. After dehydration and imbedding in paraffin, 3.5 µm sections were prepared. The sections were incubated with primary antibodies against insulin (Cat No. YT2357, Immunoway, Wuhan city, China), followed by HRP-conjugated secondary antibodies (Cat No. RS0002, Immunoway, Wuhan city, China). Images were captured using a fluorescence microscope (Nikon Eclipse E100; Nikon, Tokyo, Japan).

### 2.5 Western blot analysis

The Western blotting technique was applied for detecting the expression of skeletal muscle proteins, including PI3K (100 kDa, CST, United States), GLUT4 (56 kDa, CST, United States), and AKT (60 kDa, CST, United States). In brief, 100 mg of skeletal muscle was immersed in RIPA buffer for 30 min and then centrifuged at 12,000 rpm for 10 min at 4 °C. The total protein was extracted from skeletal muscle, and the concentrations were determined using a Bradford Protein Assay Kit (Beyotime, Shanghai City, China). The proteins were separated on 10% SDS-PAGE gels, followed by transfer onto nitrocellulose membranes. The blocking solution containing 5% nonfat dried milk was used for incubating the membranes for 1.5 h at room temperature. The membranes were then incubated overnight at 4 °C with the following primary antibodies: AKT (#9272), GLUT4 (#2213), and PI3 kinase (#4292), which were obtained from Cell Signaling Technology, Massachusetts, United States, each at a dilution of 1: 1,000; the GAPDH antibody was also used at a dilution of 1: 1,000. Next, the membranes were washed in TBST four times and incubated with suitable secondary antibodies for 1.5 h at room temperature. The protein bands were exposed in the chemiluminescent reagent for approximately 5–10 min after incubation with the secondary antibodies. Protein expression was detected by fluorescence captured on X-ray photographic film in a dark room. Finally, the protein band densities were quantified, and the gray level of the above proteins was calculated using ImageJ software (NIH, Bethesda, MD, United States).

## 3 Statistical analysis

All parameters in this study were calculated as the mean ± SD in each group. Statistical analyses and graphing were performed using SPSS Statistics 22.0 and GraphPad Prism 8.0 software (San Diego, CA, United States). One-way (or two-way) analysis of variance (ANOVA) was used for multiple comparisons among the four groups, followed by Bonferroni’s post hoc tests. The statistical comparisons between the two groups were analyzed using the unpaired t-test. Generally, changes were considered statistically significant (*) when *p <* 0.05.

## 4 Results

### 4.1 HPLC-MS analysis of Huoxue-Jiangtang decoction

Based on the determination results, HPLC-MS methods for HXJT were established. Quality control of all herbs was performed according to the Chinese Pharmacopoeia, and the contents of 22 components of HXJT were analyzed by HPLC-MS. Consistent separation and reproducible chromatograms were obtained, with 22 distinct peaks identified as common peaks (peak 1–22) (Figures 1A–D). Table 2 shows the quantified components of HXJT.

### 4.2 Antihyperglycemic effect of HXJT in T2DM rats

**FIGURE 2:**
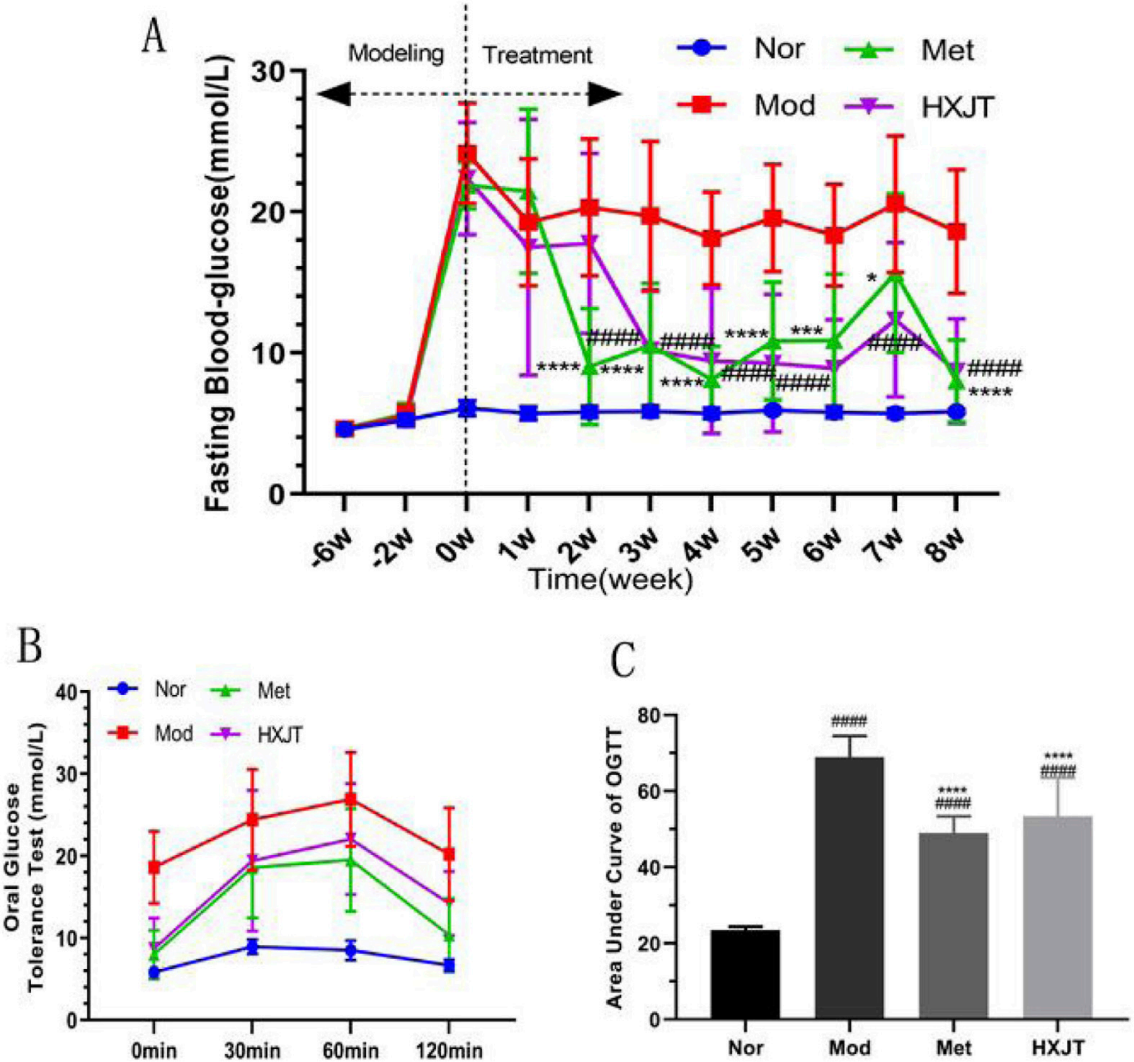
Effects of HXJT on FBG and glucose tolerance. **(A)** FBG was measured from 08:00 to 08:30 on the last day of each week. ^####^ *p <* 0.0001 vs*.* Mod; *p < 0.05, ***p < 0.001, and ****p < 0.0001 vs*.* Mod. **(B)** OGTT; the OGTT experiment was conducted on the weekend of the last week of drug administration; **(C)** AUC of OGTT; ^####^ *p <* 0.0001 vs*.* Nor; ****p < 0.0001 vs*.* Mod. The results are presented as the means ± SD, and n = 10 in each group. Nor, normal group; Mod, model; Met, metformin group; HXJT, HXJT treatment group.

We monitored FBG to assess the effects of HXJT in T2DM rats. After 6 weeks of T2DM modeling (−6w to 0w), FBG levels of modeling rats conformed to the T2DM standard (Figure 2A). After 2 weeks of treatment, the metformin-treated group (Met) showed a significant reduction in FBG levels (*p* < 0.001), while a significant effect was observed in the HXJT group (*p* < 0.001) starting from the third week; the best curative effect of metformin and HXJT appeared in the eighth week (*p* < 0.0001) (Table 3; Figure 2A). After 8 weeks of treatment, the Met and HXJT groups showed 62.78% (*p* < 0.0001) and 58.73% (*p* < 0.0001) reductions in FBG levels, respectively, compared to those in the Mod group (20.99%). The OGTT value and area under the curve of OGTT (AUC-OGTT) are shown in Table 4 and Figures 2B,C. The AUC-OGTT values in the Met (49.02) and HXJT groups (53.39) were remarkably lower (*p* < 0.001) than those in the Mod group (68.88).

**TABLE 3:** Effects of HXJT on FBG.

Time (week)	Nor	Mod	Met	HXJT
–6 week	4.57 ± 0.16	4.61 ± 0.33	4.64 ± 0.44	4.53 ± 0.25
–2 week	5.23 ± 0.39	5.51 ± 0.71	5.7 ± 0.7	5.32 ± 0.47
0 week	6.08 ± 0.53	24.15 ± 3.55^####^	21.87 ± 1.63^####^	22.36 ± 3.97^####^
1 week	5.71 ± 0.48	19.26 ± 4.5^####^	21.45 ± 5.82	17.48 ± 9.06
2 week	5.81 ± 0.41	20.31 ± 4.85^####^	9.03 ± 4.11****	17.76 ± 6.38
3 week	5.85 ± 0.38	19.72 ± 5.27^####^	10.53 ± 4.4****	10.17 ± 4.18****
4 week	5.71 ± 0.37	18.12 ± 3.29^####^	8.1 ± 2.35****	9.43 ± 5.15****
5 week	5.93 ± 0.18	19.55 ± 3.8^####^	10.83 ± 4.17****	9.26 ± 4.86****
6 week	5.77 ± 0.39	18.33 ± 3.6^####^	10.9 ± 4.68***	8.91 ± 3.44****
7 week	5.72 ± 0.31	20.55 ± 4.84^####^	15.65 ± 5.62*	12.34 ± 5.46****
8 week	5.84 ± 0.15	18.6 ± 4.39^####^	8.01 ± 2.9****	8.72 ± 3.69****

Data are described as the mean ± SD (n = 9).

^####^ *p* < 0.0001 vs*.* Nor; *p < 0.05, ***p < 0.001, and ****p < 0.0001 vs*.* Mod. Nor, normal group; Mod, model; Met, metformin group; HXJT, HXJT, treatment group.

**TABLE 4:** Effects of HXJT on glucose tolerance (OGTT) after treatment.

Time (min)	Nor	Mod	Met	HXJT
0 min	5.84 ± 0.15	18.6 ± 4.39^####^	8.01 ± 2.9****	8.72 ± 3.69****
30 min	8.95 ± 0.86	24.42 ± 6.13^####^	18.58 ± 6.15*	19.42 ± 8.56
60 min	8.5 ± 1.16	26.9 ± 5.72^####^	19.5 ± 6.24*	22.05 ± 6.72
120 min	6.68 ± 0.68	20.2 ± 5.64^####^	10.36 ± 4.46***	14.22 ± 3.89*

Data are described as the mean ± SD (n = 9).

^####^ *p* < 0.0001 vs*.* Nor; *p < 0.05, ***p < 0.001, and ****p < 0.0001 vs*.* Mod.

### 4.3 Ameliorative effect of HXJT on insulin resistance in T2DM rats

**FIGURE 3:**
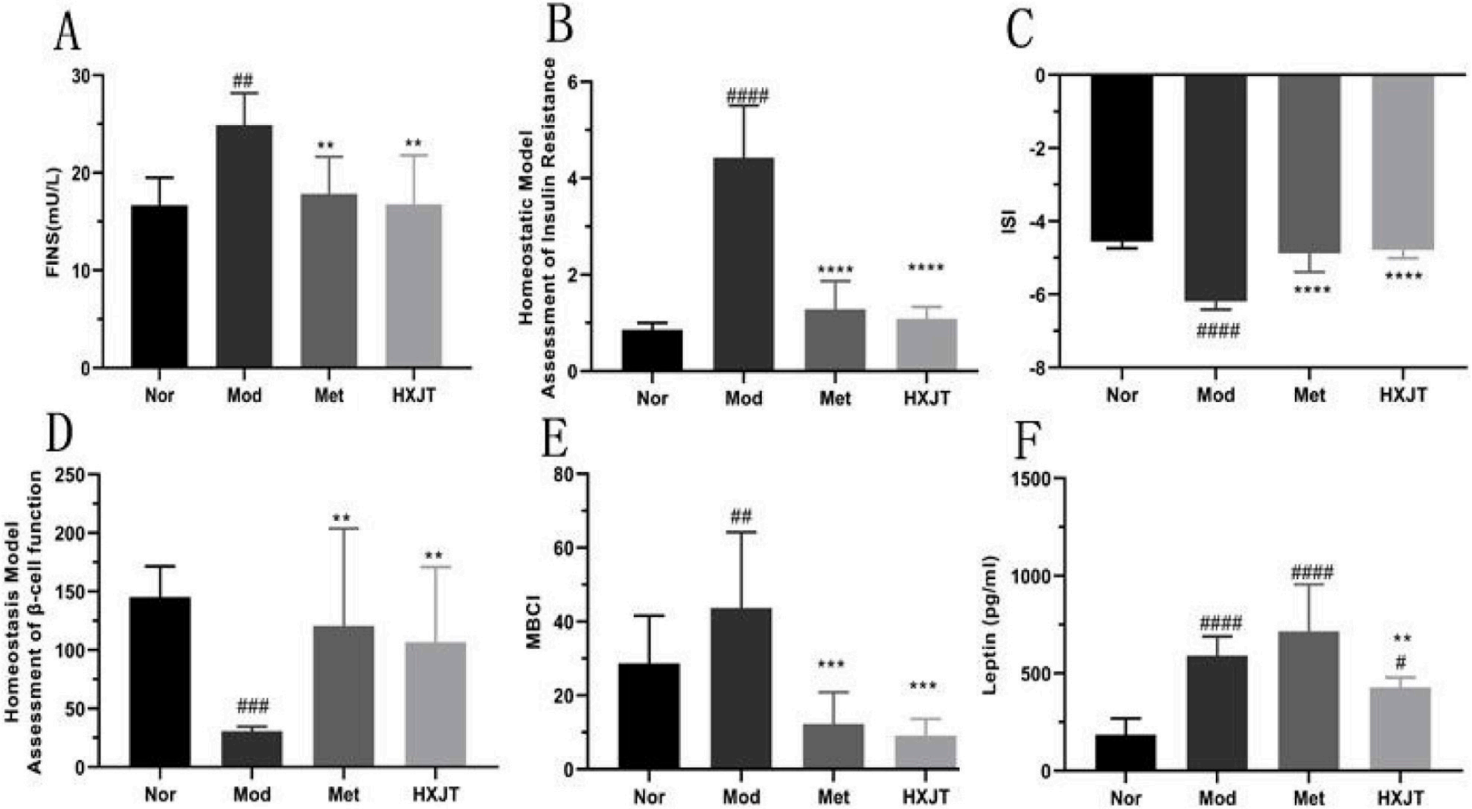
Effects of HXJT on fasting serum insulin (FINS) levels, homeostasis model assessment of insulin resistance (HOMA-IR), insulin sensitivity index (ISI), homeostasis model assessment- β (HOMA-β), and modified β-cell function index (MBCI). FINS **(A)**, HOMA-IR index **(B)**, ISI **(C)**, HOMA-β **(D)**, MBCI **(E)**, and **(F)** leptin were detected in each group after 8 weeks of treatment. ^#^ *p <* 0.05, ^##^ *p <* 0.01, ^###^ *p <* 0.001, and ^####^ *p <* 0.0001 vs*.* Nor; **p < 0.01, ***p < 0.001, and ****p < 0.0001 vs*.* Mod. The results are presented as the means ± SD, and n = 8 in each group. Nor, normal group; Mod, model; Met, metformin group; HXJT, HXJT treatment group.

Figure 3A indicates that the FINS level of the Mod group (24.88 ± 3.30 mU/L) was significantly higher than that of the Nor group (16.68 ± 2.83 mU/L) after 8 weeks of administration (*p* < 0.01). However, compared with that in the Mod group, the FINS level of the Met (17.83 ± 3.84 mU/L, *p* < 0.01) and HXJT groups (16.80 ± 5.03 mU/L, *p* < 0.01) was significantly reduced after 8 weeks of treatment. The HOMA-IR level of the Mod group was strikingly higher than that in the Nor group (*p* < 0.0001), and in both Met (1.29 ± 0.58, *p* < 0.0001) and HXJT groups (1.09 ± 0.25, *p* < 0.0001), the HOMA-IR level was remarkably lower than that in the Mod group (4.43 ± 1.08) (Figure 3B). As shown in Figure 3C, the level of ISI was markedly decreased in the Mod group compared with that in the Nor group (*p* < 0.0001). Interestingly, the FBG levels in the Met and HXJT groups were markedly reduced, while ISI was increased compared with that in the Mod group (*p* < 0.0001). Moreover, the HOMA-β level in both Met and HXJT groups was markedly increased (*p* < 0.01) compared with that in the Mod group after 8 weeks of treatment (Figure 3D), and the MBCI level was also significantly reduced (*p* < 0.001) in the two treatment groups (Figure 3E). Moreover, the leptin level in the Mod group was drastically higher than that in the Nor group (*p* < 0.0001); however, it significantly decreased in the HXJT group (*p* < 0.01) (Figure 3F).

### 4.4 Ameliorative effect of HXJT on lipid metabolism in T2DM rats

**FIGURE 4:**
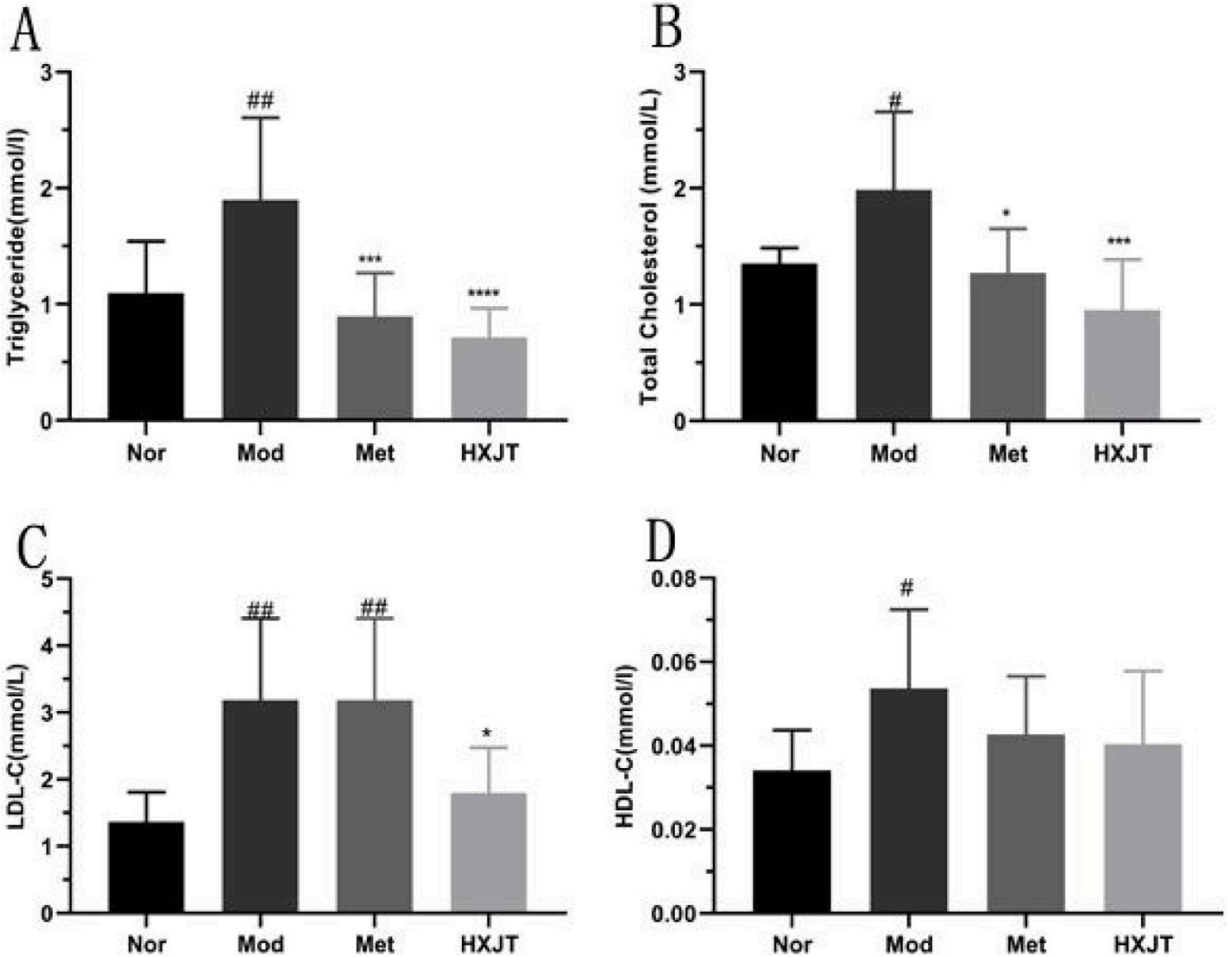
Effect of HXJT on lipid metabolism in T2DM rats. **(A)** Triglycerides (TGs), **(B)** total cholesterol (TC), **(C)** low-density lipoprotein cholesterol (LDL-C), **(D)** high-density lipoprotein cholesterol (HDL-C) levels. The total TC, TG, HDL-C, and LDL-C levels in blood serum were measured in each group after treatment. The results are presented as the means ± SD, and n = 9 in each group. ^#^ *p <* 0.05 and ^##^ *p <* 0.01 vs*.* Nor; *p < 0.05, ***p < 0.001, and ****p < 0.0001 vs*.* Mod. Nor, normal group; Mod, model; Met, metformin group; HXJT, HXJT treatment group.

To analyze the effects of HXJT on lipid metabolism, the blood lipid profile, including TC, TG, HDL-C, LDL-C and FFA, was measured, as shown in Figures 4A–D. The Mod group displayed significant dyslipidemia compared with the Nor group (*p* < 0.05), while both the Met and HXJT groups reversed the serum TG and TC to close to normal levels (*p* < 0.05). The HXJT group could alleviate the LDL-C level (*p* < 0.05), while the Met group did not change compared to the Mod group. In addition, the Met and HXJT groups reduced FFA levels to some extent, although the differences were not statistically significant compared with the Mod group (Supplementary Figure S2D).

### 4.5 Histopathological changes and insulin expression in pancreatic tissue

**FIGURE 5:**
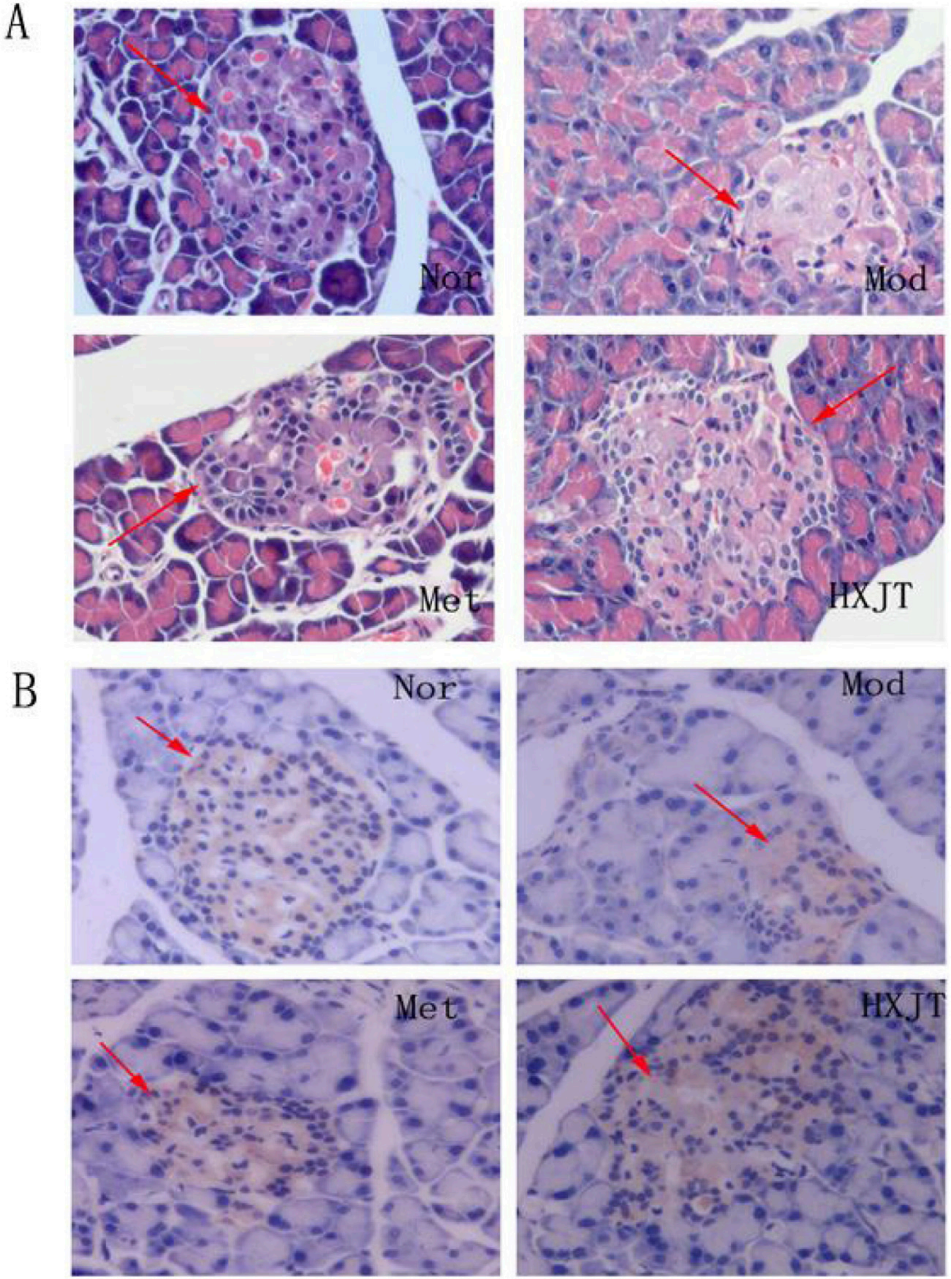
Effect of HXJT on the morphology of pancreatic tissues. Representative images of hematoxylin and eosin (HE) staining **(A)** (×400, n = 4) and insulin expression in the pancreas [immunohistochemistry, **(B)**] in the tails of pancreas from each group of mice after 8 weeks of treatment (×400, n = 4). Nor, normal group; Mod, model; Met, metformin group; HXJT, HXJT treatment group.

The paraffin sections of the pancreatic tissue were stained with HE (Figure 5A). As expected, significant differences in the number and pattern of the islets were observed among the four groups. The Mod group showed islets with extensive vacuolated areas, whereas the islets in both treatment groups appeared round and well-defined. These results suggested that HXJT and metformin could restore STZ-induced islet damage, with our findings indicating that HXJT further enhances the recovery of islets in T2DM rats.

The borders of the pancreas islets were distinctive from those of the exocrine glands because of adequate zymogen granules. Immunohistochemical analysis showed insulin expression in pancreatic islets, with the granules appearing brown-stained.

### 4.6 Effects of HXJT on AKT, GLUT4, and PI3K expressions in skeletal muscles

**FIGURE 6:**
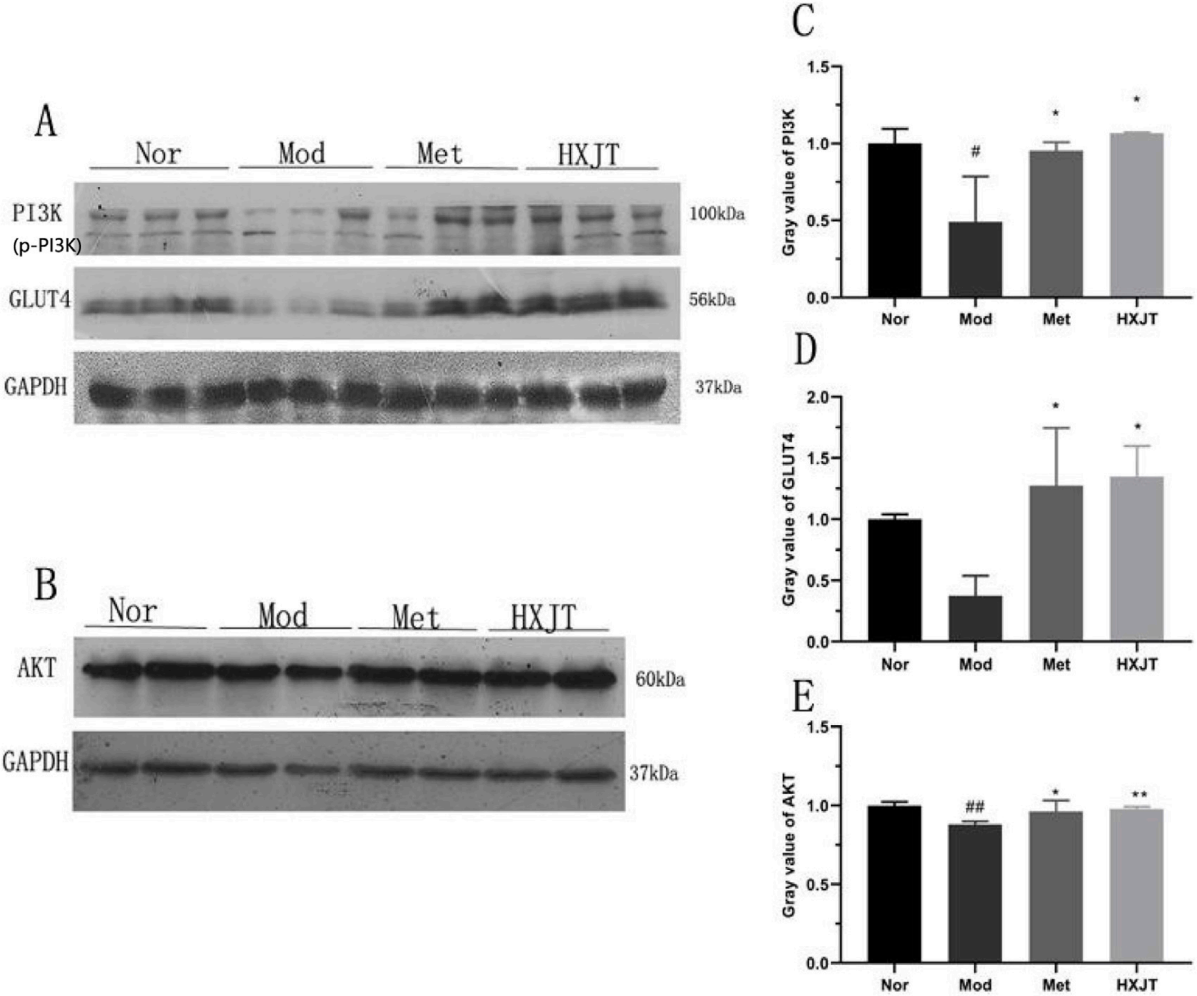
Effects of HXJT on AKT, PI3K, and GLUT4 expressions in skeletal muscle through Western blot analysis. **(A, C, D)** Representative expressions of PI3K and GLUT4 in skeletal muscle. Data are presented as the mean ± SD (n = 3). **(B, E)** Expression of AKT in skeletal muscles. Data are presented as the mean ± SD (n = 4). ^#^ *p <* 0.05 and ^##^ *p <* 0.01 vs*.* Nor; *p < 0.05 and **p < 0.01 vs*.* Mod. Nor, normal group; Mod, model; Met, metformin group; HXJT, HXJT treatment group.

To assess whether HXJT reduced blood glucose levels and improved insulin resistance through the PI3K/AKT signaling pathway or by regulating GLUT4 protein expression, the levels of AKT, PI3K, and GLUT4 in skeletal muscles were detected using Western blot (Figures 6A–E). As shown in Figure 6, the PI3K, GLUT4, and AKT levels were significantly reduced in the Mod group compared with those in the Nor group (*p* < 0.05 or *p* < 0.01). As shown in Figure 6A, the band corresponding to p-PI3K is presented directly below the PI3K band. It can be visually confirmed that the expression level of p-PI3K in the HXJT group is significantly higher than that in the model (Mod) control group. The Met- and HXJT-treated groups significantly reversed the downregulation of AKT and PI3K expression compared with the Mod group (*p* < 0.05 or *p* < 0.01).

### 4.7 Effects of HXJT on glycogen content and oxidative stress in T2DM rats

**FIGURE 7:**
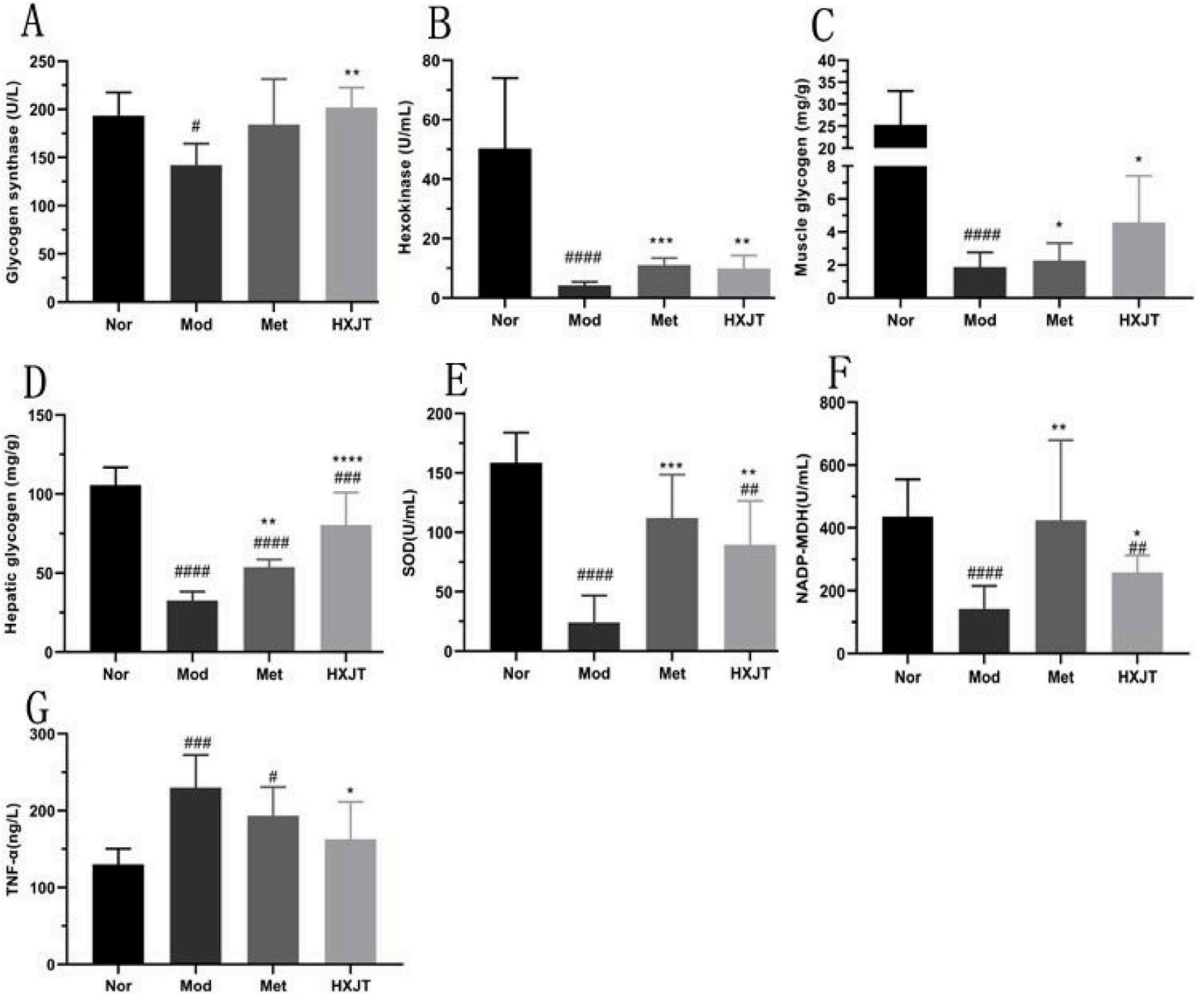
Effects of HXJT on glycogen, TNF-α, and some key metabolic enzymes. **(A)** Glycogen synthetase (GS), **(B)** hexokinase, **(C)** muscle glycogen in skeletal muscle, **(D)** hepatic glycogen in the liver, **(E)** activity of superoxide dismutase (SOD), **(F)** NADP-malic dehydrogenase (NADP-MDH), and **(G)** TNF-α. Data are presented as the mean ± SD (n = 8). ^#^ *p <* 0.05, ^##^ *p <* 0.01, ^###^ *p <* 0.001, and ^####^ *p <* 0.0001 vs*.* Nor; *p < 0.05,**p < 0.01, ***p < 0.001, and ****p < 0.0001 vs*.* Mod. Nor, normal group; Mod, model; Met, metformin group; HXJT, HXJT treatment group.

To evaluate the effects of HXJT on glycogen content, the glycogen content, glycogen synthase, hexokinase, NADP-malic dehydrogenase, hepatic glycogen in the liver, and muscle glycogen in skeletal muscle were measured. As shown in Figures 7A–D,F, glycogen synthase, hexokinase, muscle glycogen, hepatic glycogen, and NADP-malic dehydrogenase levels in the HXJT group were remarkably increased compared to those in the Mod group (*p <* 0.05).

To explore the alleviation effect of HXJT on oxidative stress in T2DM rats, SOD and TNF-α levels were detected. The TNF-α levels in the Met and HXJT groups were notably decreased compared to those in the Mod group (*p* < 0.05), while the SOD level was significantly increased (*p* < 0.01).

### 4.8 Effect of HXJT on the body weight and liver enzyme index

The body weight gain of the four groups is shown in Supplementary Figure S1A, without a significant difference (*p* > 0.05) among the four groups. We monitored food consumption (kcal/) and water consumption weekly (mean), as shown in Supplementary Figure S1B,C; both had no significant statistical differences (*p* > 0.05) between the Mod group and the other two administered groups. ALT and AST were the main liver enzyme indices; abnormal liver enzymes were often used as an important biological indicator of abnormal liver function (Gowda et al., 2009). In the blood biochemical tests, the results of serum ALT and AST are described in Supplementary Figure S2A,B, without statistical differences (*p* > 0.05) among the four groups; this suggests to some extent that HXJT may possess relatively low hepatotoxicity, while other potential toxic effects will be further elucidated in future studies.

## 5 Discussion

T2DM is mainly characterized by insulin resistance, often associated with obesity, oxidative stress, and inflammation, along with impaired insulin secretion that ultimately leads to disordered glucolipid metabolism. Because of its complications and pathogenesis affect numerous systems, it is difficult to expect a single-target medicine to treat T2DM. As expected, herbal medicine therapies act on multiple targets, which may help address this metabolic disorder once their comprehensive and effective mechanisms are fully elucidated (Loizzo et al., 2016; Vinayagam et al., 2017).

Therefore, the present study focused on evaluating the efficacy of HXJT in T2DM rats as it has shown promising therapeutic effects in patients with T2DM in China. As shown by HPLC-MS analysis in Table 2, HXJT contains 22 contents identified phytochemical features. As expected, astragaloside IV (the extracts of *Astragalus membranaceus*) (Xu et al., 2018), catalpol (extract from Rehmanniae Radix) (Huang et al., 2010), and amygdalin (extract from Semen Persicae) (Heikkila and Cabbat, 1980), which are the main components of HXJT, have significant effects on ameliorating blood glucose and lipid levels. The HFD and STZ-induced T2DM rat model was widely used for T2DM research (Skovso, 2014). In the present study, the T2DM rats displayed evidently higher FBG levels (>11.1 mmol/L) before treatment (0w) (Lu et al., 2016) (Table 3; Figure 2A), poorer OGTT (Figures 2B,C), more serious insulin resistance (Figure 3), higher blood lipid profile levels (Figure 4), and serious pancreatic damage (Figure 5) than the normal rats. The FBG, HOMA-IR, and pathological results of T2DM rats indicated that the T2DM rats developed comparable symptoms to human T2DM. All these suggested that the composite model for T2DM was produced successfully. After treatment with HXJT, the results of the HXJT group showed that HXJT treatment could lower FBG, improve glucose tolerance, and activate the insulin signaling pathway (Figure 6).

Insulin resistance and islet function are the main characteristics of T2DM. Therefore, improving insulin resistance and islet function is a key strategy for the treatment and prevention of T2DM. In our study, metformin and HXJT reduced the FINS level at a rate of 28.33% and 32.15%, respectively, compared to that in the Mod group (Figure 3A). The HOMA-IR and MBCI levels evidently reduced (Figures 3B,E), while the HOMA-β and ISI levels remarkably increased, suggesting that HXJT and metformin could ameliorate insulin resistance in T2DM rats. In addition, both HXJT and metformin could repair the damage of the pancreatic islets that was induced by HFD and STZ (Figures 5A,B). Moreover, the T2DM rats had a higher leptin level, and HXJT could reduce the leptin level. Obesity is associated with leptin production and high serum leptin concentration (Considine et al., 1996). HFD could induce leptin resistance (Koch et al., 2014); this appearance would be consistent with insulin resistance in our study. HXJT treatment reduced leptin resistance and increased leptin sensitivity. The adiponectin level in the HXJT group also had an upward trend, although there were no statistical differences (Supplementary Figure S2C). Generally, although HXJT and metformin share similarities in their hypoglycemic effects and ability to improve insulin resistance, HXJT differs from metformin in other indices, likely due to their different mechanisms of action.

In T2DM rats, a high-fat diet could lead to lipid metabolism disorders (Yan et al., 2016). Our results indicated that the levels of these blood lipid indices (TG, TC, and LDL-C) were significantly downregulated after HXJT treatment. However, metformin could not significantly decrease LDL-C, which was notably higher than that with HXJT treatment, although it could reduce LDL-C in T2DM patients (Lin et al., 2018).

The PI3K pathway is related to glucose metabolism (Nandipati et al., 2017). As the previous studies suggested (Yu et al., 2017; Nana et al., 2016), the PI3K/AKT signaling pathway and GLUT4 protein were critical for insulin-induced glucose uptake in skeletal muscle. In our study, HXJT could increase the expressions of AKT and PI3K in skeletal muscle (Figures 5A–C,E); the activation of the AKT protein could motivate expression and translocation of GLUT4, resulting in increased glucose uptake. The AMPK pathway plays a central role in sustaining glucolipid metabolism (Nandipati et al., 2017), which is a crucial regulatory pathway of GLUT4 translocation, and it could enhance blood glucose uptake in skeletal muscle to improve blood glucose homeostasis (Konrad et al., 2005). Catalpol (Guangying, 2008) and hydroxyl safflower yellow A (Yueqing et al., 2017) could enhance insulin sensitivity, promote blood glucose uptake, and preserve pancreatic tissue, which might trigger GLUT4 via the PI3K/AKT signal pathways. HXJT not only increased the GLUT4 protein expression but also significantly increased the content of muscle glycogen in skeletal muscle and increased the activity of hexokinase for further improving the ability of glucose reuptake (Figures 5B,C). Moreover, in the HXJT group, the level of active glycogen synthase was higher than that in the Mod group, which could promote the synthesis of hepatic glycogen (Figures 5A,D). Taken together, our findings suggested that HXJT could enhance blood glucose uptake and lower blood glucose levels (Cline et al., 2002). The liver is a key metabolic organ, which is also the target organ of injury of T2DM (Bril et al., 2017). The serum AST and ALT levels could sensitively reflect the injury of the liver. In this study, serum levels of AST and ALT did not significantly different among the four groups (Supplementary Figure S2A,B), indicating that HXJT treatment did not cause obvious liver injury.

Finally, many tissues and organs would be damaged by hyperglycemia, which would cause the glycation reaction of antioxidant enzymes and lower the activity of SOD or other metabolic enzymes (Asmat et al., 2016). SOD affects the balance of the system of oxidation and antioxidation, which can also eliminate superoxide anion and defend tissues from damage. Malic dehydrogenase is the key enzyme in the tricarboxylic acid cycle, which plays a positive role in the metabolism of the mitochondria. Moreover, neutralization of TNF-α in obese rats caused a significant increase in the peripheral uptake of blood glucose in response to insulin (Hotamisligil et al., 1993). In our study, the TNF-α level in the HXJT group was evidently decreased compared to that in the Mod group; contrarily, the levels of SOD and NADP-malic dehydrogenase were remarkably increased (Figures 6E–G). These findings suggest that HXJT exerts anti-inflammatory and antioxidant effects to a certain extent.

## 6 Conclusion

In conclusion, we demonstrated that HXJT treatment exerts a significant anti-diabetic effect in high-fat diet- and streptozocin-induced T2DM rats, potentially improving glucose/lipid metabolism and insulin resistance. The underlying molecular mechanisms involve, at least in part, the direct or indirect modulation of critical signaling factors in the PI3K/AKT pathway and the GLUT4 protein. HXJT shows promising therapeutic potential for the treatment of T2DM.

## Data Availability

The original contributions presented in the study are included in the article/Supplementary Material; further inquiries can be directed to the corresponding authors.

## Abbreviations

HXJTD, Huoxue-Jiangtang decoction; TCM, traditional Chinese medicine; T2DM, Type 2 diabetes mellitus; HFD, high-fat diet; STZ, streptozotocin; OGTT, oral glucose tolerance test; FBG, fasting blood glucose; FINS, fasting insulin; AUC, area under the curves; TC, total serum cholesterol; TGs, triglycerides; LDL-C, low-density lipoprotein cholesterol; HDL-C, high-density lipoprotein cholesterol; HOMA-IR, homeostatic model index–insulin-resistant; HOMA-β, homeostasis model assessment of β-cell function; ISI, insulin sensitivity index; MBCI, modified β-cell function index; ALT, alanine aminotransferase; AST, aspartate aminotransferase; HPLC-MS, high-performance liquid chromatographic method–mass spectrometry.
