# Supplementary material for: Huoxue-Jiangtang decoction ameliorates type 2 diabetes in high-fat diet and streptozotocin-induced rats
Source: Front Pharmacol. 2025 Oct 17;16:1675295. doi: 10.3389/fphar.2025.1675295 (PMC12575354; doi:10.3389/fphar.2025.1675295)
Supplement: Supplementary file 1 [file DataSheet1.pdf]

Supplementary Material

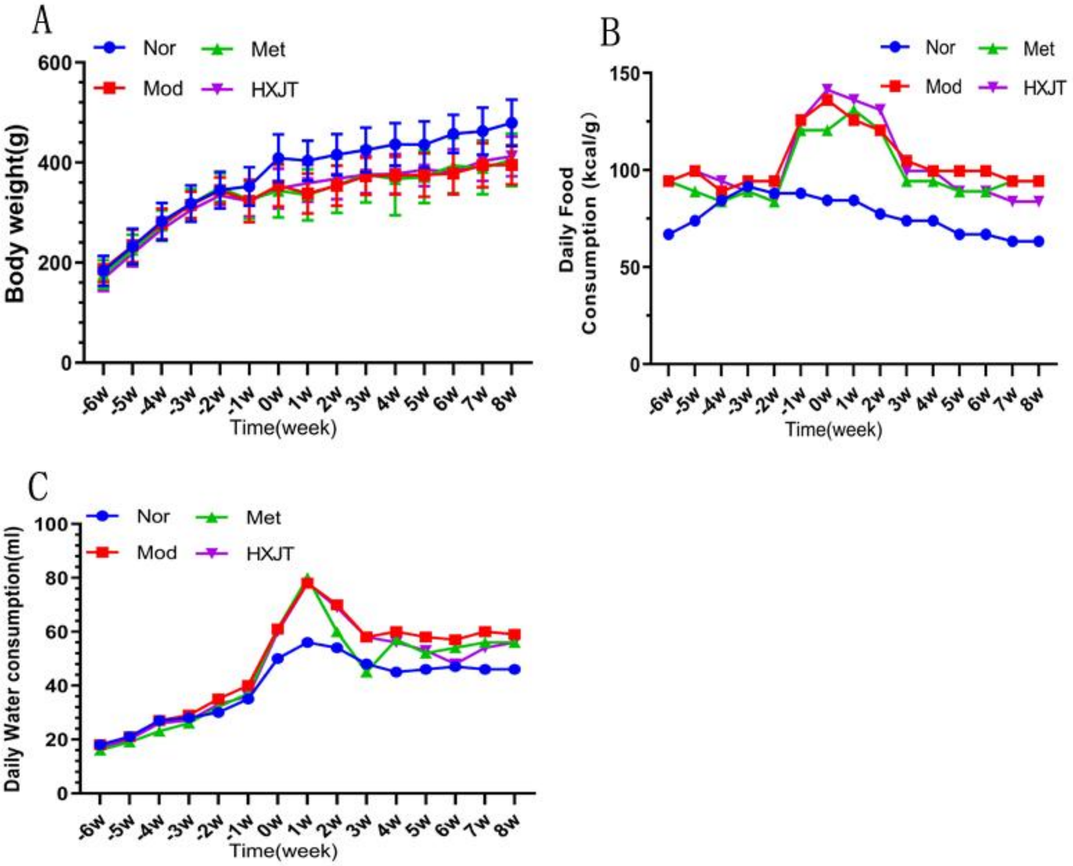

Supplementary Figure 1. Effects of HXJT on body weight(A), daily food consumption (kcal/g) (B) and daily water Consumption (ml) (C). The time point: modelling time (-6w-0w), treatment time (0w-6w). Nor: normal group; Mod: model; Met: metformin group; HXJT: HXJT treatment group.

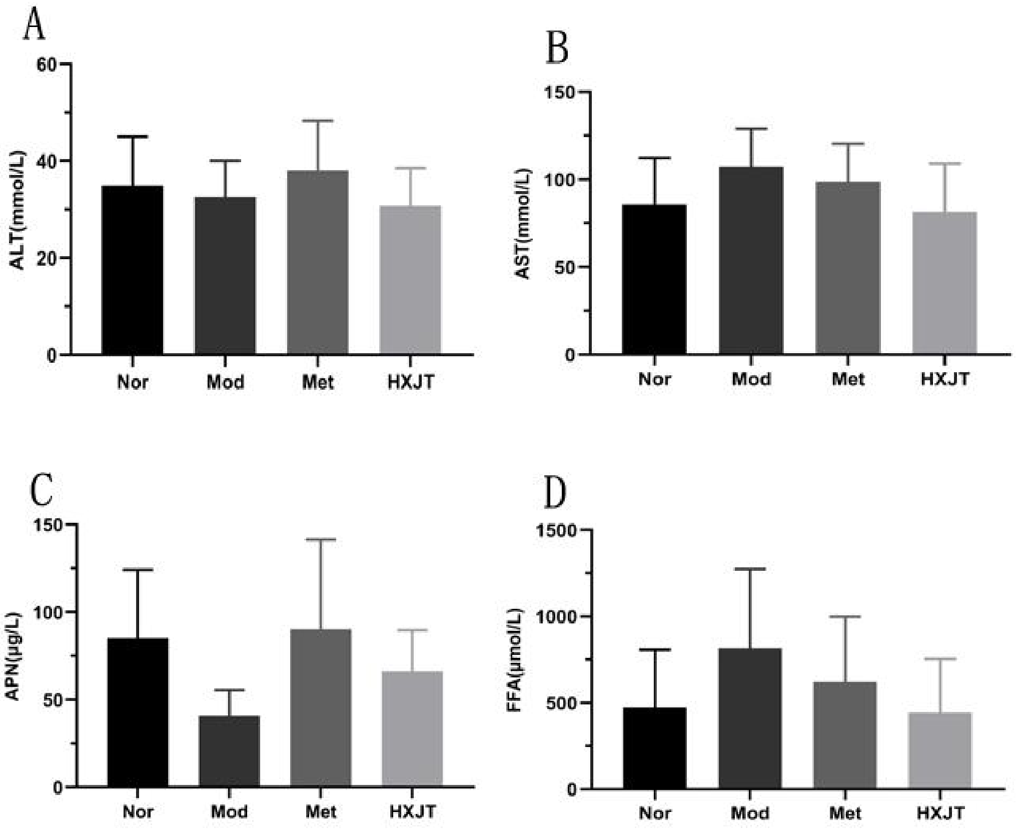

Supplementary Figure 2. Effects of HXJT on aspartate aminotransferase (AST)(**A**) ,alanine aminotransferase (ALT)(**B**), adiponectin (APN)(**C**) and free fatty acids (FFA)(**D**). All above index have no statistical difference mong the four groups. Nor: normal group; Mod: model; Met: metformin group; HXJT: HXJT treatment group.

Supplementary Table 1. High -fat diet (D12492)

Product	D12492	
	**gm%**	**kcal%**
Protein	26.2	20
Carbohydrate	26.3	20
Fat	34.9	60
**Total (kcal/gm)**	5.24	
**Ingredient**	**gm**	**kcal**
Casein,80 Mesh	200	800
L-Cystine	3	12
Corn Starch	0	0
Maltodextrin 10	125	500
Sucrose	68.8	275.2
Cellulose, BW200	50	0
Soybean Oil	25	225
Lard*	245	2205
Mineral Mix, S10026	10	0
Dicalcium Phosphate	13	0
Calcium Carbonate	5.5	0
Potassium Citrate, 1 H2O	16.5	0
Vitamin Mix, V10001	10	40
Choline Bitartrate	2	0
FD&C Blue Dye #1	0.05	0
**Total**	**773.85**	**4057**

*Typical analysis of cholesterol in lard = 0.95 mg/gram.
Cholesterol (mg)/4057 kcal = 232.8
Cholesterol (mg)/kg = 300.8

Supplementary Table 1. The formula of the High -fat diet was specified in detail in this table.
